# *Gymnosiphon syceorosensis* (Burmanniaceae), the second new species for the Philippines

**DOI:** 10.3897/phytokeys.146.48321

**Published:** 2020-05-08

**Authors:** Daniel L. Nickrent

**Affiliations:** 1 Department of Plant Biology, Southern Illinois University, Carbondale, 62901-6509, Illinois, USA Southern Illinois University Carbondale United States of America

**Keywords:** Dioscoreales, Mindanao, monocot, Mt. Hamiguitan, mycoheterotroph

## Abstract

A new holomycoheterotrophic member of Burmanniaceae, *Gymnosiphon syceorosensis*, is described from Mt. Hamiguitan located on the island of Mindanao, Philippines. This species differs from the recently named *G. philippinensis* from Cebu in a number of quantitative and qualitative characters. Phenetic (neighbor-joining) and phylogenetic (maximum parsimony) analyses of characters from Asian and Australian *Gymnosiphon* species were conducted and diagnostic taxonomic features were discussed. This new species appears to be most closely related to *G. affinis* J.J. Sm. from New Guinea but differs in a number of floral features including inner perianth lobe shape, stamen position in floral tube, and anther connective shape.

## Introduction

Mycoheterotrophs are plants that obtain nutrients from mycorrhizal fungi that are attached to the roots of vascular plants as well as from saprophytic fungi (Leake 1994). This trophic form occurs in ten angiosperm families, some of which are green and photosythetic and are called partial mycoheterotrophs whereas others have little or no photosynthetic activity and are called full mycoheterotrophs (Merckx et al. 2013). These conditions are analagous to hemi- and holoparasitic angiosperms. Because both life forms can be found in various genera of Burmanniaceae (Dioscoreales) and even among different species of *Burmannia*, this family provide an opportunity to study the evolutionary origins of these trophic modes. Among the eight genera in the family (Merckx et al. 2013; Burmanniaceae s. str., i.e. without five genera now placed in Thismiaceae), *Campylosiphon* Benth., *Burmannia* L. and *Gymnosiphon* Blume have both Neo- and Paleotropical members. The holomycoheterotrophic genus *Gymnosiphon* was listed by Merckx et al. (2013) as containing 16 Neotropical, 8 African + Madagascan, and 9 Asian species. Since that publication, two species have been added: *G. philippinensis* Pelser, Salares & Barcelona (Philippines) and *G. queenslandicus* Gray, Mahyuni & Low (Australia) bringing the total number of species to 35 (Gray et al. 2019; Pelser et al. 2019). As pointed out by Pelser et al. (2019), most Asian species are rarely collected, reflecting either true rarity or the fact that these plants are often overlooked.

Field work on the Philippine island of Mindanao was conducted during June 2019 as part of a project funded by the National Science Foundation entitled “Plant Discovery in the Southern Philippines”. One excursion included the Mount Hamiguitan Range Wildlife Sanctuary, a UNESCO World Heritage site that contains many Philippine endemic and endangered plant species such as *Nepenthes copelandii* Merr. ex Macfarl., *Paphiopedilum adductum* Asher, *Rhododendron kochii* Stein, and *Shorea polysperma* Merr. (Amoroso and Aspiras 2011). During the course of general collecting, specimens initially identified as *Burmannia* were obtained. Part of this collection was later determined to be *Gymnosiphon* by the presence of an unwinged ovary with prominent locular glands, parietal placentation, and a deciduous perianth limb. Merrill (1924, p. 251) excluded *G. aphyllus* from the Philippines and no other species were listed for the archipelago. The recently named *G. philippinensis* Pelser, Salares & Barcelona (Pelser et al. 2019), collected on limestone substrate in southern Cebu, is apparently the first documentation of this genus in the Philippines (Pelser et al. 2011 onwards). Those authors concluded that this taxon represented a new species based on a manual comparison to the morphologies of other Asian *Gymnosiphon* species. A similar method was used in Gray et al. (2019) for *G. queenslandicus* Gray, Mahyuni & Low.

**Figure 1.**
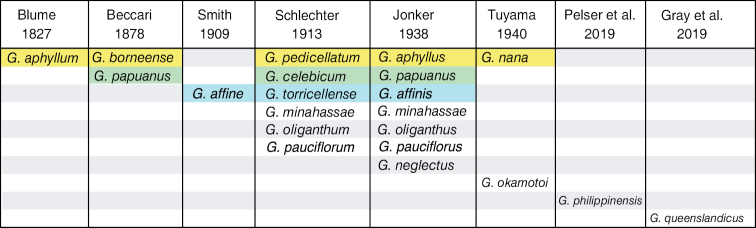
Species concepts over time in Asian and Australian *Gymnosiphon*. Names shaded with the same color represent synonyms of the same species according to Jonker (1938). The two taxa named by Tuyama (1940) were not included in this study. The synonymy of *G. nana* (*nanus*) is based upon World Plants Online (2020).

The present study presents results from cladistic and phenetic analyses that were used to assist the process of determining the distinctiveness of the Hamiguitan taxon as compared to previously described species. Moreover, these data also provided information about species boundaries in Gymnosiphon section Gymnosiphon Urb. This was deemed necessary given the different taxonomic concepts for Asian and Australian *Gymnosiphon* species as published by different authors over the past century (Fig. 1). The matrices constructed included continuous (quantitative) and categorical (qualitative) characters and these were analyzed separately and in a concatenated matrix. The separate analyses were conducted to determine if similar relationships emerged no matter what the partition or method of analysis. Ideally, these morphological data should be examined in the context of a molecular phylogeny of the species, but the only published studies of this type (Merckx et al. 2006, 2008) sampled just one Asian species (*G. aphyllus* Blume) among the 11 accessions included.

## Materials and methods

### Field work

Flowering individuals of the Mt. Hamiguitan *Gymnosiphon* taxon were photographed *in situ*. Collections were dried and pressed as herbarium vouchers (no. 1314) and tissue was dried in silica gel for later DNA extraction and sequencing. A few individuals were also placed in bottles containing 70% ethanol for later examination. Dissection and photography of the fixed tissues was accomplished with an Olympus SZH-10 stereomicroscope fitted with a Leica MC190HD digital camera.

### Character scoring

For comparisons with other Asian and Australian *Gymnosiphon* species, descriptions and illustrations from the primary literature were examined. The taxa *G. nanus* (Fukuy. & T.Suzuki) Tuyama from Orchid Island and *G. okamotoi* Tuyama from Republic of Palau were not included because their protologues were obtained after manuscript submission. From these a list of characters that appeared taxonomically useful was compiled. The original observations for this study as well as information from the literature were compiled in an Excel spreadsheet (Suppl. material 1: Table S1). These were mainly from (Jonker 1938, 1948) and Schlechter (1913) as well as the descriptions of *G. aphyllus* by Beccari (1878) and Smith (1922). Three species (*G. minahassae* Schltr., *G. oliganthus* Schltr., and *G. pauciflorus* Schltr.) were only collected once thus the taxon descriptions of Schlechter and Jonker (who examined the same specimens) were consolidated. For *G. affinis* J.J.Sm., *G. aphyllus* Blume and *G. papuanus* Becc., the descriptions and taxonomic views differed among Blume, Schlechter and Jonker, thus, they were considered separately (see Fig. 1).

Twelve continuous (i.e. quantitative) and 12 categorical (i.e. qualitative, discrete) characters were used (Tables 1, 2, respectively). Schlechter (1913) provided both quantitative and qualitative characters in his descriptions, albeit not consistently for all structures and species. Photographs of the six types used by Schlechter are available for examination at BGBM (Berlin). These images have sufficient resolution to allow some characters to be scored, e.g. lengths of scale leaves, flowers, and fruits. Some data were obtained for quantitative characters not explicitly mentioned in the literature by extrapolating from drawings. This was justified because the sizes of some structures (e.g. flower length) mentioned in the articles could be confirmed from the herbarium specimen image. From the type photos, measurements were taken from as many structures as possible and the mean values recorded. The use of original author descriptions to generate the categorical characters posed some difficulties because each employed different terminology. The following is a brief listing of the continuous (0–11) and categorical (12–23) characters used in this study. For additional discussion of these characters, see Suppl. material 2: File S2.

**Table 1.** Continuous characters 0–11 for *Gymnosiphon* taxa used in this study. Top line is currently accepted name, bottom line is source of descriptive data and in some cases synonyms. The top number represents the ln-transformed standardized range (0 to 10), the bottom number the observed range. Missing data are shown as “?”.

Taxa/Characters	0	1	2	3	4	5	6	7	8	9	10	11
*G. affinis* J.J. Sm.	5.170	2.369	4.531	7.112	3.479	5.532	0.000	0.000	?	0.000	3.856	2.609
(Jonker 1938)	2.398	0.916	1.099	1.253	1.946	1.253	0.916	0.470	?	0.916	1.447	1.099
*G. affinis* J.J. Sm.	5.170	6.915	1.123	3.673	3.479	4.950	8.480	4.563	0.988	5.305	10.000	0.000
(*G. torricellensis*Schlechter 1913)	2.398	1.118	0.693	0.693	1.946	1.194	1.504	0.924	0.668	1.131	1.668	0.956
*G. aphyllus* Blume	5.530	?	5.827	7.112	6.280	4.008	8.797	5.621	?	2.795	6.058	4.890
(*G. borneensis* Becc.)	2.442	?	1.253	1.253	2.140	1.099	1.526	1.030	?	1.030	1.526	1.224
*G. aphyllus* Blume	6.520	1.915	4.531	9.304	7.105	5.532	8.797	4.239	2.835	3.660	0.150	8.568
(*G. pedicellatum*Schlechter 1913)	2.565	0.896	1.099	1.609	2.197	1.253	1.526	0.892	0.756	1.065	1.314	1.426
*G. aphyllus* Blume	5.873	?	5.332	10.000	7.578	5.245	7.655	3.861	0.773	9.669	0.000	10.000
(Smith 1909)	2.485	?	1.194	1.723	2.230	1.224	1.447	0.854	0.658	1.308	1.308	1.504
*G. aphyllus* Blume	7.119	4.513	?	7.933	7.885	5.532	8.480	4.073	?	4.497	2.169	6.676
(Jonker 1938)	2.639	1.012	?	1.386	2.251	1.253	1.504	0.875	?	1.099	1.386	1.322
*G. minahassae* Schlechter	4.795	4.676	3.951	6.165	4.475	2.594	7.824	6.513	3.225	2.795	2.992	1.864
(Schlechter 1913)	2.351	1.019	1.030	1.099	2.015	0.956	1.459	1.118	0.775	1.030	1.416	1.058
*G. neglectus* Jonker	4.600	4.513	10.000	0.000	3.479	4.008	2.630	2.241	10.000	4.497	?	2.609
(Jonker 1938)	2.327	1.012	1.749	0.095	1.946	1.099	1.099	0.693	1.099	1.099	?	1.099
***G. syceorosensis* Nickrent**	4.401	5.316	4.390	2.853	3.479	4.008	0.000	0.900	4.351	5.305	2.169	4.346
(G. sp. 1314, this ms.)	2.303	1.047	1.082	0.560	1.946	1.099	0.916	0.560	0.829	1.131	1.386	1.194
*G. oliganthus* Schlechter	2.341	5.551	2.998	6.165	0.000	0.000	4.854	7.862	0.445	0.000	2.169	1.022
(Schlechter 1913)	2.048	1.058	0.916	1.099	1.705	0.693	1.253	1.253	0.642	0.916	1.386	1.012
*G. papuanus* Becc.	4.401	4.513	5.204	0.000	4.475	2.206	10.000	7.862	?	0.000	6.951	4.068
(Jonker 1938)	2.303	1.012	1.179	0.095	2.015	0.916	1.609	1.253	?	0.916	1.558	1.179
*G. papuanus* Becc.	6.203	10.000	3.800	0.000	3.479	0.761	6.415	5.657	2.238	0.967	2.652	0.755
(*G. celebicum*Schlechter 1913)	2.526	1.256	1.012	0.095	1.946	0.770	1.361	1.033	0.728	0.956	1.404	0.997
*G. pauciflorus* Schlechter	1.519	0.000	5.827	0.000	5.406	1.164	10.000	10.000	0.224	4.497	3.856	2.116
(Schlechter 1913)	1.946	0.811	1.253	0.095	2.079	0.811	1.609	1.466	0.631	1.099	1.447	1.072
*G. philippinensis* Pelser et al.	0.000	9.284	6.844	2.853	5.406	4.950	7.485	4.031	5.332	8.298	8.379	1.991
(Pelser et al. 2019)	1.758	1.224	1.374	0.560	2.079	1.194	1.435	0.871	0.875	1.253	1.609	1.065
*G. queenslandicus* Gray et al.	2.598	6.469	1.533	1.906	4.475	3.326	7.991	5.799	0.000	4.497	2.169	2.609
(Gray et al. 2019)	2.079	1.099	0.742	0.405	2.015	1.030	1.470	1.047	0.621	1.099	1.386	1.099
*G. suaveolens* (H.Karst) Urb.	10.000	6.469	0.000	9.304	10.000	10.000	5.850	0.187	1.202	10.000	9.736	7.853
(Maas-van de Kamer 1998)	2.996	1.099	0.560	1.609	2.398	1.705	1.322	0.489	0.678	1.322	1.658	1.386

**Table 2.** Categorical characters 12–23 for *Gymnosiphon* taxa used in this study. Top line is currently accepted name, bottom line is source of descriptive data and in some cases synonyms. Missing data are shown as “?”.

Taxa/Characters	12	13	14	15	16	17	18	19	20	21	22	23
*G. affinis* J.J. Sm.	0,1	?	0	?	1	0	3	1	1	0	1	0
(Jonker 1938)												
*G. affinis* J.J. Sm.	0	0	2	0	1	0	3	2,3	0	1	1	0
(*G. torricellensis*Schlechter 1913)												
*G. aphyllus* Blume	1	?	2	?	1	?	0, 1	0, 1	0	?	0	0
(*G. borneensis* Becc.)												
*G. aphyllus* Blume	1	3	0	1	1	1	2	1	0	3	0	0
(*G. pedicellatum*Schlechter 1913)												
*G. aphyllus* Blume	0, 1	3, 4	0	1	1	?	1	1	0	3	0	0
(Smith 1909)												
*G. aphyllus* Blume	0,1	?	0	?	1	1	0, 1	0	0	1	0	0
(Jonker 1938)												
*G. minahassae* Schlechter	0	3	2	1	0	1	1	1	0	?	0	0
(Schlechter 1913)												
*G. neglectus* Jonker	2	3	2	0	1	0	0	0	1	3	0	0
(Jonker 1938)												
***G. syceorosensis* Nickrent**	1	1	0	0	1	1	4	2	0	3	0	0
(G. sp. 1314, this ms.)												
*G. oliganthus* Schlechter	0	0	2	1	1	1	4	1, 2	0	1	0	0
(Schlechter 1913)												
*G. papuanus* Becc.	0, 1	?	0, 2	?	0	1	0	?	1	1, 2	1	0
(Jonker 1938)												
*G. papuanus* Becc.	1	0	0, 2	0	0	1	0	1	1	2	1	0
(*G. celebicum*Schlechter 1913)												
*G. pauciflorus* Schlechter	0	1	2	0	0	1	0	1	1	2	0	0
(Schlechter 1913)												
*G. philippinensis* Pelser et al.	1	2	1	1	0	0	0	0	1	0	1	0
(Pelser et al. 2019)												
*G. queenslandicus* Gray et al.	1	0	0	0	1	1	3	3	1	0	0	0
(Gray et al. 2019)												
*G. suaveolens* (H.Karst) Urb	1	4	0	0	0	1	4	1	1	2	0	1
(Maas-van de Kamer 1998)												

0 Plant height (cm);

1 Leaf length (mm);

2 Floral bract length (mm);

3 Pedicel length (mm);

4 Flower length (mm);

5 Outer perianth lobe length (mm);

6 Floral tube length (mm);

7 Ratio floral tube to outer perianth lobe length;

8 Ratio outer perianth lobe length to width;

9 Ovary length (mm);

10 Fruit length (mm);

11 Persistent floral tube length (mm);

12 Inflorescence type: (0) simple cyme, (1) bifid cyme, (2) capitate;

13 Outer perianth lobe outline including marginal lobes: (0) orbicular, (1) broadly ovate, (2) ovate, (3) rectangular, (4) broadly obtrulate;

14 Outer perianth lobe outline without lateral lobes: (0) ovate, (1) narrowly ovate, (2) triangular;

15 Outer perianth lobe margin to apex: (0) below apex, (1) equal apex;

16 Outer perianth lobe margin: (0) entire, (1) crenate;

17 Outer perianth lobe color: (0) white, (1) violet;

18 Inner perianth lobe shape: (0) linear, (1) lanceolate, (2) ovate, (3) obovate, (4) cuneate;

19 Inner perianth lobe apex: (0) acute, (1) obtuse, (2) truncate, (3) 3-lobed;

20 Position of stamens in floral tube: (0) just below inner perianth lobe, (1) between inner perianth lobe and ovary;

21 Connective shape: (0) quadrangular, (1) triangular, (2) forked, (3) elliptic;

22 Connective apiculate: (0) no, (1) yes;

23 Stigma appendages: (0) no, (1) yes.

### Analyses

Sizes reported in the literature as ranges were converted to median values. Means were calculated from original observations from the Hamiguitan samples as well as measurements taken from the BGBM photographs. Data matrices containing the untransformed data were constructed in Mesquite (Maddison and Maddison 2018) and exported as Nexus and TNT (Tree Analysis using New Technology) files for downstream analyses (Goloboff et al. 2008; Suppl. material 3: File S3). The categorical characters were used “as is” in later analyses whereas the continuous character mean values were natural log-transformed [ln(x+1)] and range-standardized [x_s_ = (x – min/max – min) × 10] as outlined in Thiele (1993) with Microsoft Excel. All characters were treated as unordered.

Uncorrected distances for the transformed continuous character matrices were generated using Mesquite. Neighbor-joining (NJ) was performed separately on this matrix and the “as is” categorical character matrix using PAUP* (Swofford 2002). Maximum parsimony (MP) analyses of the categorical data were conducted with PAUP*. The neotropical species *Gymnosiphon suaveolens* was chosen as the outgroup for all analyses because ancestral area analyses suggests the genus originated in the New World and took a boreotropical migration route to the Old World (Merckx et al. 2008). MP analyses of the continuous and concatenated (continuous plus categorical) data matrices were conducted with TNT. The log-transformed standardized continuous characters were optimized as additive with Farris (1970) optimization. The search routine as implemented in “aquickie.run” finds optimal scores 20 times independently by using defaults of “xmult” plus 10 cycles of tree-drifting (Goloboff 1999). For strict consensus calculation, TBR (tree bisection reconnection) collapsing was used (Goloboff and Farris 2001). The direction and magnitude of change for the continuous characters was determined by using the TNT command blength for each of the 11 characters. Bremer (1994) support values (decay indices) were calculated which represent the difference (number of steps) between the score of the most parsimonious tree and the next most parsimonious tree where the node in question is lost.

## Results and discussion

**Figure 2.**
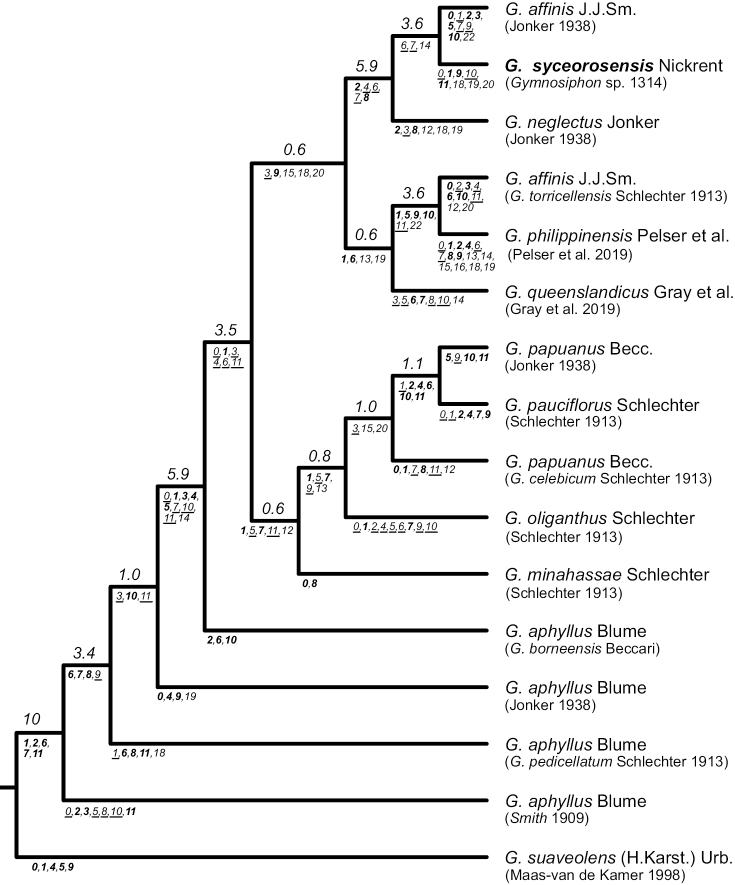
Maximum parsimony cladogram derived from the concatenated continuous and categorical data matrices. Below the currently accepted taxon names are the sources of descriptive data and in some cases synonyms (see Suppl. material 1: Table S1). Numbers above branches are Bremer support values. Numbers below the nodal branches are unambiguous synapomorphies that occurred on every tree. Characters 0–11 are continuous, 12–23 categorical. For the continuous characters, increases are shown in bold, decreases as underlined fonts. Taxon names follow Jonker (1938, 1948) plus two recently named taxa (*G. philippinensis* and *G. queenslandicus*).

The MP strict consensus tree from the concatenated continuous and categorical data matrices analyzed with TNT (Fig. 2) contains clades with varying degrees of support as measured by Bremer decay index values. The four *Gymnosiphon aphyllus* terminals are present as a grade at the base of the tree. A well-supported clade (Bremer decay index > 5 steps) is composed of *G. aphyllus* (*G. borneensis* Beccari) and all remaining species. A clade composed of *G. minahassae*, *G. oliganthus*, *G. pauciflorus* and the two *G. papuanus* terminals is present, but with a Bremer index of < 1 step. Bremer support was higher (> 3 steps) for the sister relationship between *G. affinis* (*G. torricellensis* Schlechter) and *G. philippinensis*. This clade was then sister to *G. queenslandicus*, but with lower Bremer support. Finally, a well-supported clade (> 5 steps) was recovered containing *G. neglectus*, *G. affinis* Jonker and *Gymnosiphon* sp. 1314.

The above results can be compared to those obtained when the continuous and categorical characters are analyzed separately using phenetic and MP methods (Suppl. material 4: File S4). A number of relationships shown in Fig. 2 are recovered as clusters (Suppl. material 4: File S4A) or clades (Suppl. material 4: File S4B) when the continuous characters are analyzed separately. These include the grade of *G. aphyllus*, the clade of *G. papuanus* Becc. and *G. pauciflorus*, the clade *G. affinis* (*G. torricellensis*) and *G. philippinensis*, and the clade of the new taxon *Gymnosiphon* sp. 1314 with *G. affinis* J.J. Sm. and *G. neglectus*. The taxa *G. minahassae*, *G. oliganthus*, and *G. papuanus* (*G. celebicum* Schlechter) emerged as a grade in the TNT analysis (Fig. 2) but as a cluster or clade (Suppl. material 4: File S4A, B, respectively) when continuous characters were analyzed alone. Analysis of the categorical characters separately (Suppl. material 4: File S4C, D) resulted in topologies that differed substantially from the TNT results (Fig. 2).

Overall, it appears that a greater contribution to the tree shown in Fig. 2 came from the continuous, not categorical characters. Both NJ and MP analyses of this data partition recovered similar groupings as compared with the concatenated dataset analyzed with MP in TNT. When apomorphies are plotted on the strict consensus tree, the majority (60%) are autapomorphic (confined to the terminals). Of the 66 synapomorphies, only 11 are categorical characters. This study demonstrates that greater resolution can be obtained by including continuous characters, which has been shown in empirical studies (e.g. Hardy et al. 2008) as well as simulations (Parins-Fukuchi 2017).

The type species for the genus, *Gymnosiphon aphyllus*, may be the earliest diverging member among the Asian species. The variation in descriptive data (Suppl. material 1: Table S1) probably results from different taxonomic terminology and interpretation of the morphology as well as real polymorphism that exists among populations of this widespread taxon. The first explanation can be demonstrated by comparing the descriptions of Schlechter and Jonker who both examined the same specimens. The four *G. aphyllus* terminals did not form a clade with MP or a cluster with NJ in any of the analyses. Jonker (1938) lumped *G. borneensis* Beccari and *G. pedicellatus* Schlechter into *G. aphyllus* Blume (Fig. 1). Because of their weak association, further study of original material from all of these taxa is required to justify any decision regarding lumping or splitting. Because species boundaries cannot easily be determined from morphology alone, a molecular phylogenetic study of all these taxa is required.

The clade composed of *G. minhassae*, *G. oliganthus*, *G. papuanus* and *G. pauciflorus* (Fig. 2) has nearly the same composition as the group formed from NJ of continuous characters (Suppl. material 4: File S4A), with the exception that the latter contains *G. queenslandicus*. *Gymnosiphon pauciflorus* is sister to *G. papuanus* and that clade sister to *G. papuanus* Becc. that was considered *G. celebicum* by Schlechter (Fig. 2). To avoid a paraphyletic *G. papuanus*, one could lump *G. pauciflorus* into *G. papuanus* or recognize three species. *Gymnosiphon pauciflorus* shares several features with *G. papuanus* (Suppl. material 1: Table S1), including characters states such as entire outer perianth lobe margins, linear inner perianth lobes, the position of stamens in the floral tube and forked connectives. Differences that were used in the key by Jonker include the number of flowers in the inflorescence, a meristic character not used here because of extreme variation and overlap. For these two taxa, Jonker (1938) indicates “3-many” for *G. papuanus* and “1–3” for *G. pauciflorus*. Because the latter was collected only once (Schlechter 16653), this taxon could represent a few-flowered variant of *G. papuanus*. Interestingly, the species nearest to *G. pauciflorus* in the Jonker key was *G. neglectus* that occurs in a distant clade in Fig. 2.

Jonker (1938) combined *G. torricellensis* with *G. affinis*, describing the type of the former as “incomplete material but very probably belonging to this species.” With reference to *G. torricellensis*, Schlechter (1913) wrote (translated from German): “Of all the species hitherto known from the monsoon area, the present one is well differentiated by the broad, slightly three-lobed petals, and by the anthers.” The description in that work was complete (Suppl. material 1: Table S1) and analysis of this taxon with the description of *G. affinis* from Jonker (1938) results in the two being present in two different clades (Fig. 2). This result was consistent across all partitions and analytical methods. The two taxa differ in several taxonomic characters including outer perianth lobe margin, inner perianth lobe shape and apex, position of stamen in floral tube, connective shape, and ratio of floral tube to outer perianth lobe length. For this reason, it seems prudent to maintain these two taxa as different species. The taxon *Gymnosiphon okamotoi* Tuyama was not included in these analyses; however, after examining its description and illustration (Tuyama 1940), it is clear this species has strong affinity with *G. affinis* and may even be conspecific with it.

The results of the present study agree with the assessment by Gray et al. (2019) that *Gymnosiphon queenslandicus* is closely related to *G. affinis* s. lat., which is reflected with the categorical but not the continuous characters. Although *G. torricellensis* occurs together with *G. philippinensis* and *G. queenslandicus*, Bremer support for that clade is relative low. Gray et al. (2019), who examined one of the type collections of *G. affinis* (Versteeg 1425) noted that the position of the stamens was illustrated incorrectly in Smith (1909) and that they actually occur below the middle of the floral tube. This character state also occurs in *G. neglectus* and *G. philippinensis* but not *G. torricellensis* and *Gymnosiphon* sp. 1314. Whether this feature changes through floral developmental (bud through anthesis) should be investigated, although for *G. affinis*, Jonker (1938 p. 31) says that even in very young buds there is still “lowly” insertion of the stamens.

*Gymnosiphon* sp. 1314 is clearly not conspecific with *G. philippinensis* because it differs in many character states such as outer perianth lobe margin, flower color, inner perianth lobe shape, position of the stamens in the floral tube, and length of the floral tube relative to the outer perianth lobe. NJ and MP of the continuous characters and the combined data analyses place it as sister to *G. affinis* with good Bremer support, but this relationship was not seen with analyses of the categorical characters. The unique combination of character states justifies describing the Hamiguitan taxon as a new species.

### Taxonomy

#### Gymnosiphon syceorosensis

Taxon classificationPlantae

Nickrent sp. nov.

8A7A07EE-24AF-5CD8-AC18-48EF9EC38905

urn:lsid:ipni.org:names:77209567-1

Figs 3 , 4

##### Type.

Philippines. Davao Region, Davao Oriental Province, Municipio San Isidro, Barangay La Union, Mt. Hamiguitan Range Wildlife Sanctuary, 6°43.819'N, 126°10.757'E, elev. 1184 m, 18 June 2019, *Plants & Lichens of the Southern Philippines Survey* no. 1314 (holotype: BRIT, isotypes: CMUH, SIU).

##### Diagnosis.

Similar to *G. affinis* J.J. Sm. s. str. but differing in the outer perianth lobe color (white and violet vs. pure white), inner perianth lobe shape (cuneate vs. obovate), stamen position in floral tube (just below inner lobe vs. below middle of perianth), connective shape (elliptical vs. quadrangular), and connective apex (not apiculate vs. apiculate).

**Figure 3.**
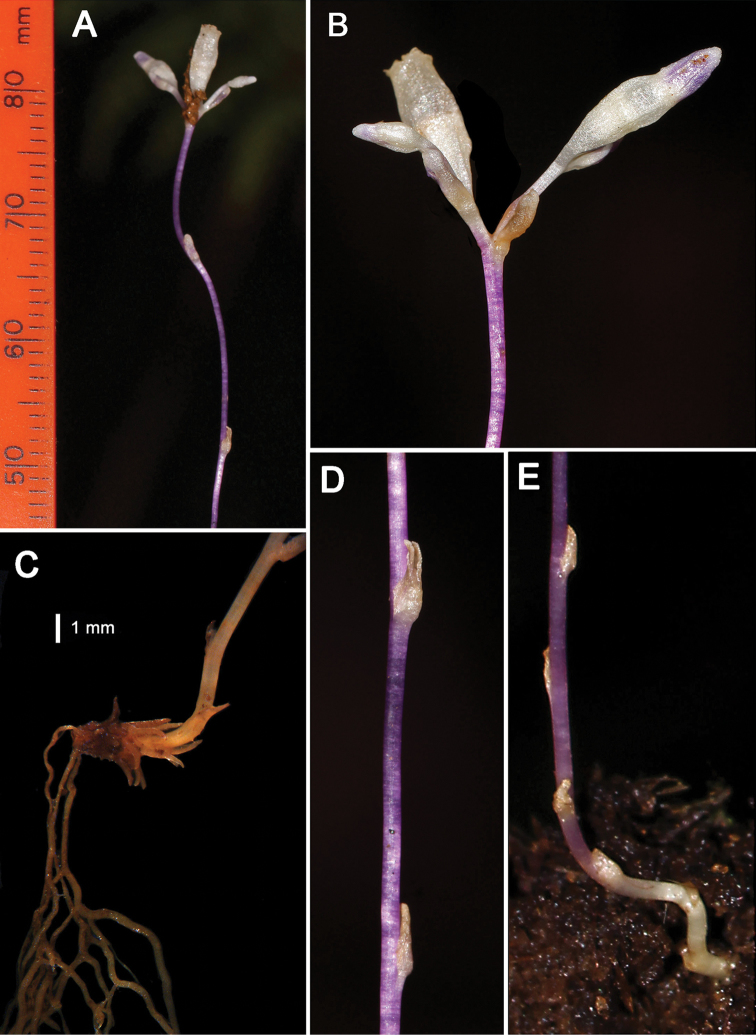
*Gymnosiphon syceorosensis* sp. nov. **A** upper portion of the plant with a young fruit in the central position of the bifid cyme. The entire plant was ca. 10 cm high **B** closer view of the flower buds and young fruit **C** underground portion of the plant (fixed in alcohol) showing short rhizome with scale leaves, exogenous roots, and basal part of aerial stem **D** closer view of stem scale leaves **E** base of aerial stem where it emerges from the soil. Photos **A, B, D, E** by Michael Galindon. Photo **C** by DLN.

##### Description.

Erect holomycoheterotrophic herb 5–10 cm tall, glabrous, achlorophyllous. Rhizome below ground, horizontal, cylindrical, 2–6 mm long, ca. 1.0 mm wide, with few short branches, covered in numerous patent, subulate scale leaves, 1–2 × 0.2–0.3 mm. Roots highly branched, contorted, 0.05–0.2 mm in diameter, lacking root hairs. Stems solitary or with a few basal branches, erect, purple, terete, 0.5 mm wide, internodes 0.3–1.5 cm long. Scale leaves sparse, spiral on stem, sessile, appressed, light tan, narrowly ovate, 1.5–2.2 mm long, base clasping ca. half the stem circumference, apex acute. Inflorescence terminal, bicincinnate (biparous cymose), terminal prophyll with two branches, each branch (peduncle) ca. 2.5 mm long, two-flowered, monochasial. Flowers erect, actinomorphic, mature buds ca. 6.0 mm long. Pedicel up to 1.0 mm long, floral bracts broadly ovate, 1.8–2.1 mm long, entire, apex obtuse. Outer perianth lobes (limbs) 3, valvate, light purple, ca. 2.0 mm long, outline (including central and lateral lobes) broadly ovate, central lobes narrowly ovate, apex acute, cucullate, lateral lobes induplicate in bud, not reaching apex of central lobe, margins somewhat crenate, wavy, undulate; floral tube white, 1.5 mm long, 1.5 mm wide, slightly constricted at junction with limbs; limbs circumscissile, caducous, separating from the top of the floral tube which persists on the fruit. Inner perianth lobes 3, inserted just below limb sinuses, cuneate, slightly folded lengthwise, ca. 0.3 mm long, apex truncate, mucronate. Anthers essentially sessile, inserted ca. 0.2 mm below insertion of inner perianth lobes, bilocular, tetrasporangiate, quadrangular in outline, ca. 0.7 mm wide; connectives narrowly elliptical in face view, projecting slightly above apex of thecae. Style cylindrical, ca. 1.8 mm long (including stigma), apical portion 3-lobed, ca. 0.7 mm wide, style branches ca. 0.3 mm long; stigma lobes hollow, funnelform, narrowly cordate (compressed laterally), ca. 0.2 mm wide, edge thickened, covered in minute papillae, apex lacking appendages. Ovary infundibuliform, ca. 2.1 mm long, 1.5 mm wide at apex, unilocular with three parietal placentae each bearing at their apices a prominent, spherical, 0.4 mm-wide gland. Fruit (immature) ca. 3.0 mm long (ovary portion), persistent floral tube cylindrical, ca. 2.3 mm long, bearing the remains of the stigmas and anthers.

**Figure 4.**
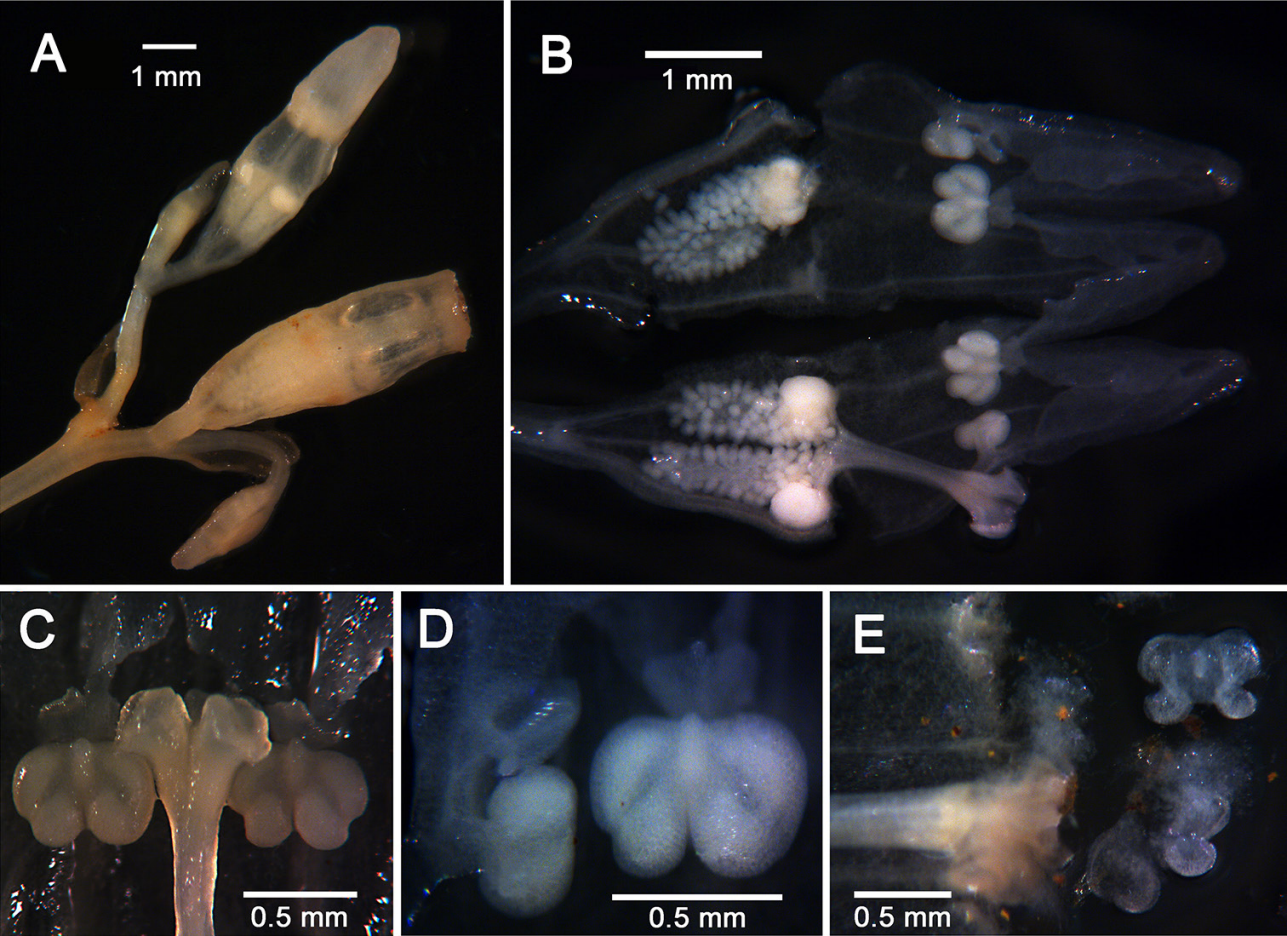
*Gymnosiphon syceorosensis* sp. nov. **A** bifid cyme (bicincinnate) showing older flower bud at top and young fruit below **B** fixed flower bud, sectioned longitudinally **C** closer view of the stigma flanked by two anthers **D** anther in longitudinal section (left) and in face view (right) showing position relative to inner perianth lobes **E** terminal portion of floral tube that is persistent on the fruit. Note the disintegrating stigma and anthers among the debris. All photos by DLN.

##### Distribution, habitat, and conservation.

*Gymnosiphon syceorosensis* is only known from the type collected in the tropical upper montane rainforest of Mt. Hamiguitan, Mindanao. The plant was found along the trail at 1184 m elevation, ca. 1 air km south of the summit of Mt. Hamiguitan. The substrate was predominantly ultramafic. This forest has the highest number of endemic and threatened plant species among the five vegetation types surveyed by Amoroso and Aspiras (2011). The habitat where this plant was found also contained other mycoheterotrophs such as *Burmannia lutescens* (a new record for this species for the Philippines) and *Sciaphila* sp. (Triuridaceae). Association of different mycoheterotrophs in one local area was mentioned by Schlechter (1913) and Pelser et al. (2019). This phenomenon may reflect the ecological requirements of the fungi or the association of different plant species with one fungus (Maas-van de Kamer 1998). The latter seems to be supported for Burmanniaceae where that family as well as Gentianaceae and Triuridaceae have been found associated with Glomerales and Diversisporales (Hynson and Bruns 2010). Because only one population of *G. syceorosensis* was discovered, no estimation of its abundance or overall distribution can me made. It, like most *Gymnosiphon* species, is likely rare in nature, but because it is inconspicuous, it is likely undercollected. Until further work can be undertaken to determine how many populations of *G. syceorosensis* exist, the conservation status of this species should at this time be considered Data Deficient (DD) according to the IUCN (2019). Note that the DD category does not imply that the taxon is not threatened.

##### Etymology.

The specific epithet commemorates the Mt. Hamiguitan Range Wildlife Sanctuary. The word “hagímit” is Cebuano for “a small tree of primary forest with rough leaves: *Ficus* sp.” (Wolff 1972). Apparently the “g” and “m” consonants were switched (a common occurrence in Cebuano), thereby producing “hamigit”. Adding the suffix “-an” which mean “a place of” gives hamigitan, i.e. “a fig tree place” or “a place with a fig tree”. When constructing the specific epithet for *Gymnosiphon*, the goal was to express “from fig-mountain”. Fig-tree is translated to Latin as “syce” (συκη, feminine) and mountain as “oros” (όρος, masculine), thus giving “syceoros” (Stearn 1992). Using one of the recommended adjectival endings for geographic epithets with a masculine termination yields “syceorosensis”.

It should be pointed that generic names derived from Greek that end in “-on” are often interpreted as neuter, however, according to ICN Art. 62.2, compound generic names take the gender of the last word in the nominative case in the compound. In this example, the Greek word element -siphon (σίφων) is masculine, thus the gender for all specific epithets of *Gymnosiphon* should be masculine. The type species was originally published by Blume (1827) as *G. aphyllum* (neuter), but this should be corrected to *G. aphyllus* (masculine).

## Supplementary Material

XML Treatment for Gymnosiphon syceorosensis

## Supplementary materials

Supplementary material 1 Original morphological data derived from the literature and, for *Gymnosiphon syceorosensis*, from original observations Data type statistical data https://binary.pensoft.net/file/407798This dataset is made available under the Open Database License (http://opendatacommons.org/licenses/odbl/1.0/). The Open Database License (ODbL) is a license agreement intended to allow users to freely share, modify, and use this Dataset while maintaining this same freedom for others, provided that the original source and author(s) are credited.Author: Daniel L. Nickrent

Supplementary material 2 List of continuous and categorical characters used for the *Gymnosiphon* taxa, with discussion Data type statistical data https://binary.pensoft.net/file/407799This dataset is made available under the Open Database License (http://opendatacommons.org/licenses/odbl/1.0/). The Open Database License (ODbL) is a license agreement intended to allow users to freely share, modify, and use this Dataset while maintaining this same freedom for others, provided that the original source and author(s) are credited.Author: Daniel L. Nickrent

Supplementary material 3 TNT matrix used in this study with 12 continuous and 12 categorical characters Data type statistical data https://binary.pensoft.net/file/407800This dataset is made available under the Open Database License (http://opendatacommons.org/licenses/odbl/1.0/). The Open Database License (ODbL) is a license agreement intended to allow users to freely share, modify, and use this Dataset while maintaining this same freedom for others, provided that the original source and author(s) are credited.Author: Daniel L. Nickrent

Supplementary material 4 Trees resulting from continuous and categorical characters analyzed separately using neighbor-joining (NJ) and maximum parsimony (MP) methods Data type statistical data Explanation note **File S4A.**NJ tree of continuous characters. **File S4B.**MP cladogram of continuous characters. **File S4C.**NJ tree of categorical characters. **File S4D.**MP cladogram of categorical characters. https://binary.pensoft.net/file/407801This dataset is made available under the Open Database License (http://opendatacommons.org/licenses/odbl/1.0/). The Open Database License (ODbL) is a license agreement intended to allow users to freely share, modify, and use this Dataset while maintaining this same freedom for others, provided that the original source and author(s) are credited.Author: Daniel L. Nickrent
