# Supplementary material for: Gymnosiphon syceorosensis (Burmanniaceae), the second new species for the Philippines
Source: PhytoKeys. 2020 May 8;146:71–87. doi: 10.3897/phytokeys.146.48321 (PMC7228931; doi:10.3897/phytokeys.146.48321)
Supplement: Supplementary material 2 — List of continuous and categorical characters used for the Gymnosiphon taxa, with discussion [file phytokeys-146-071-s002.pdf]

Supplemental File 2
Characters Used in this Study

The following 12 continuous characters are self explanatory
0. Plant height (cm)
1. Leaf length (mm)
2. Floral bract length (mm)
3. Pedicel length (mm)
4. Flower length (mm)
5. Outer perianth lobe length (mm)
6. Floral tube length (mm)
7. Ratio floral tube to outer perianth lobe length
8. Ratio outer perianth lobe length to width
9. Ovary length (mm)
10. Fruit length (mm)
11. Persistent floral tube length (mm)

The following 12 categorical characters and discussion.

12. Inflorescence type

0 simple cyme (including 1 and 3 flowered)
1 bifid cyme
2 capitate (of contracted cymes)

There are relatively few illustrations that show the inflorescence types in these Asian species (e.g. *Gymnosiphon aphyllus, G. pauciflorus*, and *G. neglectus*). Only *G. neglectus* is scored as "capitate", thus it is autapomorphic. Both simple and bifid cincinni occur in *G. affinis, G. aphyllus*, and *G. papuanus* and these taxa were scored as polymorphic (0, 1).

13. Outer perianth lobe outline including marginal lobes

0 orbicular
1 broadly ovate
2. ovate
3 rectangular
4 broadly obtrulate

This character and #14 were constructed in an attempt to capture the complex shape of these perianth lobes that are composed of two parts: the central lobe and the lateral (marginal) lobe. Looking at various descriptions, sometimes it is clear that the term refers to the outline of the entire structure whereas other times it seems best applied to just the central lobe (without margins). The original descriptive wording was left in Supplemental file 1 to illustrate this point. Actual photographs of flowers were used for *Gymnosiphon suaveolens*, *G. queenslandicus*, and *G. syceorosensis* whereas all others used illustrations. Ideally, photos or living material of all these species, at full anthesis, are required to allow a robust comparison, but these are not available. Admittedly, geometric morphometrics (statistical shape analysis such as Procrustes analysis) would be a better method than the one employed here.

14. Outer perianth lobe outline without lateral lobes

0 ovate
1 narrowly ovate
2. triangular

This captures the shape of the central lobe. The same methods as #13 were used.

15. Outer perianth lobe margin to apex

0 below apex
1 equal apex

For some taxa, the lateral lobe extends as far as the apex of the central lobe whereas in others it is below the apex. This character is an attempt to capture the "lobing" aspect.

16. Outer perianth lobe margin

0 entire
1 crenate (including undulate)

This character attempts to distinguish entire lateral lobe margins from other states. It is understood that true crenate does not equal undulate (i.e. wavy in another plane), but distinguishing between these two from drawings is difficult. Moreover, some lateral lobes appear to have a combination of a crenate and undulate in their margins.

17. Outer perianth lobe color

0 white
1 violet (including bluish, lilac, mauve)

Only taxa with pure white perianths were scored "0". For taxa that have mixtures of white and some blue, lilac, mauve, etc.), they were scored "1". There are also taxa that show polymorphism with pure white forms and colored forms in different populations (e.g. *Gymnosiphon suaveolens*).

18. Inner perianth lobe shape

0 linear (including subulate, filiform)
1 lanceolate
2 oval
3 obovate (including oblong)
4 cuneate (including obcuneate, spatulate)

Given the small size of this structure, obtaining reliable and acurate information about its shape from the literature proved challenging. There is also variation among authors' descriptions (e.g. *Gymnosiphon aphyllus*).

19. Inner perianth lobe apex

0 acute
1 obtuse
2 truncate
3 3-lobed

The description in Beccari (1878) for *Gymnosiphon borneensis* (= *G. aphyllus*) used acute but the illustration shows it as capitate. Acute agrees with Jonker (1938) but not Smith (1922) who scores it as obtuse, as does Schlechter (1913) for *G. pedicellatum* (= *G. aphyllus*). Oy vey!

20. Position of stamens in floral tube

0 just below inner perianth lobe

1 between inner perianth lobe and ovary

In the key by Jonker (1938), this character is described as “anthers inserted below the middle of the perianth” vs. “anthers inserted in the middle of the perianth”. Here, perianth refers to the ENTIRE perianth, i.e. outer perianth lobe (limb) plus the floral tube. If these two structures are of equal length, then the first character state places the anthers at their junction and “below the middle” places the anthers at some position downward in the floral tube. Confusion could occur if the “above and below middle” was misinterpreted to mean within the floral tube. The character states used here hopefully make this scoring clearer. No attempt was made to distinguish how far down the floral tube the stamens were inserted. As stated in the text, it is not known whether this feature changes through floral developmental (bud through anthesis), but here is assumed to be stable given the statement by Jonker (1938 p. 31) who said that even in very young buds there is still “lowly” insertion of the stamens in *Gymnosiphon affinis*. When looking at the distribution of this character state on the parsimony tree (Fig. 1), *G. toricellensis* and *G. syceorosensis* (=*G.* sp1314) are homoplasious (state 0) among mainly state 1 taxa.

21. Connective shape

0 quadrangular

1 triangular (including deltoid)

2 forked (including 2-shanked)

3 elliptic (including subovate)

Because this structure is very small and its 3-dimensional structure can only be ascertained by carefull dissection, the descriptions for its shape likely contain some uncertainty. This is particularly true for *Gymnosiphon minahassae*.

22. Connective apiculate (including mucronulate)

0 no

1 yes

The presence of this feature for some taxa seems straightforward, e.g. *Gymnosiphon affinis* and *G. philippinensis* both have a mucro at the apex of the connective. And the absence of the mucro in other species (*G. aphyllus*, *G. oliganthus*) is clear from the descriptions and illustrations. For the remaining species, the presence or absence of the mucro is not clear and requires a discussion of the overall construction of the anther and connective.

For *Gymnosiphon papuanus*, Jonker (1938, p. 135) did not observe anther structure directly but supposedly used the description in Schlechter (1913) stating “connective deltoid, at the top 3-lobed, with a forked thickening”. The actual Latin description in Schlechter (1913) was “Antherae brevissime stipitatae loculis basi ampliatis, connectivo incrassatione bicruri donato, apice minute trilobulato, loculos vix excedente”, i.e. “Anthers shortly stipitate at the enlarged basal locules, connective endowed with two thickened segments [legs], apex minutely trilobed, scarcely exceeding the locules.” The German description says “Antheren ziemlich weit unterhalb der inneren Segmente inseriert, sehr kurz gestielt, mit nach unten verbreiterten Fächen. Konnektiv mit zweischenkliger Verdickung, an der Spitze sehr kurz 3-lappig”, i.e. “Anthers are inserted quite far below the inner segments, very short stalked, with widened locules below. Connective with two-shanked thickening, at the apex very shortly 3-lobed.” The drawing (Fig. 1,

mislabeled C1, should be D1) shows a forked connective with tissue in the middle that is apiculate; these structures are here interpreted to be the connective.

For *G. pauciflorus*, Jonker (1938) said “connective not apiculate, forked at the top (ex Schlechter).” The Latin description in Schlechter (1913) was: “Antherae subsessiles circuitu quadratae, apice bifidae, loculis sese tingentibus”, i.e. “Anthers subsessile, square in outline, apex bifid, locules ***tingentibus***(?).” The German description says “Antheren fast sitzend, quadratisch, an der Spitze zweispaltig, von den inneren Segmenten entfernt inseriert”, i.e. Anthers almost sessile, square, bifid at the apex, inserted remotely from the inner segments.” The illustration (Fig. 1 D) is difficult to interpret. For both *G. papuanus* and *G. pauciflorus*, a tetrasporangiate nature of the anther cannot be easily seen as drawn because each theca appears to have only one locule.

Gymnosiphon syceorocensis was scored as lacking the connective mucro; however, when looking at Figs. 3C and D, one might score this species the other way. In face view, the connective in this taxon is narrowly elliptic and its apex, that projects slightly above the locules, may appear mucronate. The anther morphology in *G. syceorocensis* is most similar to that seen in *G. aphyllus* and *G. oliganthus*.

23. Stigma appendages

0 no

1 yes

None of the ingroup (section *Eugymnosiphon*) have stigma appendages which are only present in members of section *Appendiculati* Jonk. This character was added to accommodate the outgroup *G. suaveolens*.
