# Supplementary material for: Gymnosiphon syceorosensis (Burmanniaceae), the second new species for the Philippines
Source: PhytoKeys. 2020 May 8;146:71–87. doi: 10.3897/phytokeys.146.48321 (PMC7228931; doi:10.3897/phytokeys.146.48321)
Supplement: Supplementary material 3 — TNT matrix used in this study with 12 continuous and 12 categorical characters [file phytokeys-146-071-s003.pdf]

Supplemental File 3
TNT Matrix Used in This Study

```
nstates cont;
xread

24 16

&[continuous]
G_suaveolens 10.0 6.469 0.0 9.304 10.0 10.0 5.85 0.187 1.202 10.0 9.736 7.853
G_affinis_Jonker 5.17 2.369 4.531 7.112 3.479 5.532 0.0 0.0 ? 0.0 3.856 2.609
G_affinis_torr_Schlechter 5.17 6.915 1.123 3.673 3.479 4.95 8.48 4.563 0.988 5.305
10.0 0.0
G_aphyllus_bor_Beccari 5.53 ? 5.827 7.112 6.28 4.008 8.797 5.621 ? 2.795 6.058 4.89
G_aphyllus_ped_Schlechter 6.52 1.915 4.531 9.304 7.105 5.532 8.797 4.239 2.835 3.66
0.15 8.568
G_aphyllus_Smith 5.873 ? 5.332 10.0 7.578 5.245 7.655 3.861 0.773 9.669 0.0 10.0
G_aphyllus_Jonker 7.119 4.513 ? 7.933 7.885 5.532 8.48 4.073 ? 4.497 2.169 6.676
G_minahassae 4.795 4.676 3.951 6.165 4.475 2.594 7.824 6.513 3.225 2.795 2.992 1.864
G_neglectus 4.6 4.513 10.0 0.0 3.479 4.008 2.63 2.241 10.0 4.497 ? 2.609
G_syceorosensis 4.401 5.316 4.39 2.853 3.479 4.008 0.0 0.9 4.351 5.305 2.169 4.346
G_oliganthus 2.341 5.551 2.998 6.165 0.0 0.0 4.854 7.862 0.445 0.0 2.169 1.022
G_papuanus_Jonker 4.401 4.513 5.204 0.0 4.475 2.206 10.0 7.862 ? 0.0 6.951 4.068
G_papuanus_Schlechter 6.203 10.0 3.8 0.0 3.479 0.761 6.415 5.657 2.238 0.967 2.652
0.755
G_pauciflorus 1.519 0.0 5.827 0.0 5.406 1.164 10.0 10.0 0.224 4.497 3.856 2.116
G_philippinensis 0.0 9.284 6.844 2.853 5.406 4.95 7.485 4.031 5.332 8.298 8.379 1.991
G_queenslandicus 2.598 6.469 1.533 1.906 4.475 3.326 7.991 5.799 0.0 4.497 2.169
2.609

&[numeric]
G_suaveolens 140001411201
G_affinis_Jonker [0 1]?0?10311010
G_affinis_torr_Schlechter 0020103[2 3]0110
G_aphyllus_bor_Beccari 1?2?1?[0 1][0 1]0?00
G_aphyllus_ped_Schlechter 130111210300
G_aphyllus_Smith [0 1][3 4]011?110300
G_aphyllus_Jonker [0 1]?0?11[0 1]00100
G_minahassae 032101110?00
G_neglectus 232010001300
G_syceorocensis 110011420300
G_oliganthus 0021114[1 2]0100
G_papuanus_Jonker [0 1]?[0 2]?010?1[1 2]10
G_papuanus_Schlechter 10[0 2]001011210
G_pauciflorus 012001011200
G_philippinensis 121100001010
G_queenslandicus 100011331000
;
cc-.;
proc/;
```
