# Supplementary material for: Gymnosiphon syceorosensis (Burmanniaceae), the second new species for the Philippines
Source: PhytoKeys. 2020 May 8;146:71–87. doi: 10.3897/phytokeys.146.48321 (PMC7228931; doi:10.3897/phytokeys.146.48321)

Supplemental File 4A
Continuous Characters
NJ tree

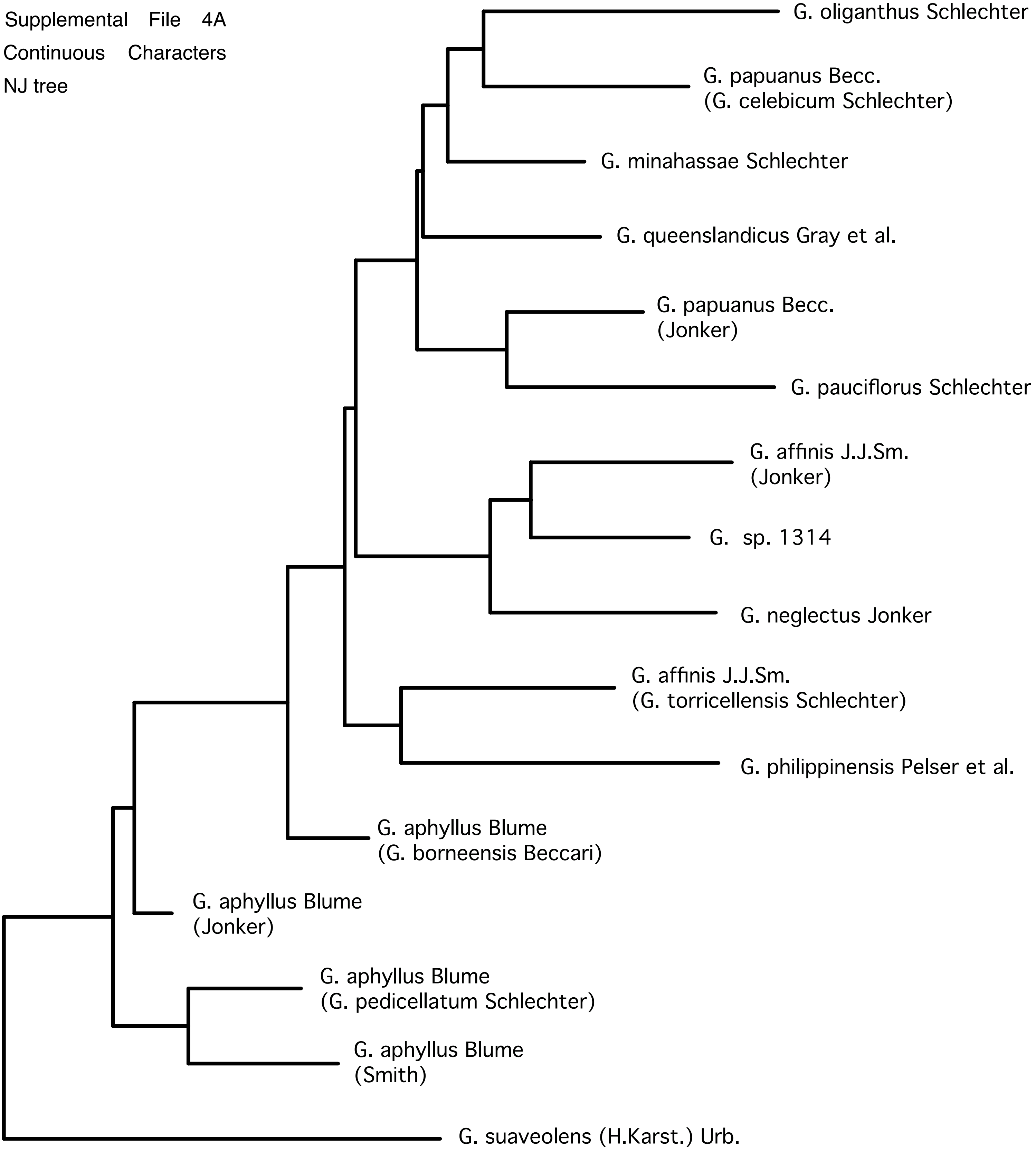

Supplemental File 4B

Continuous Characters
Maximum Parsimony

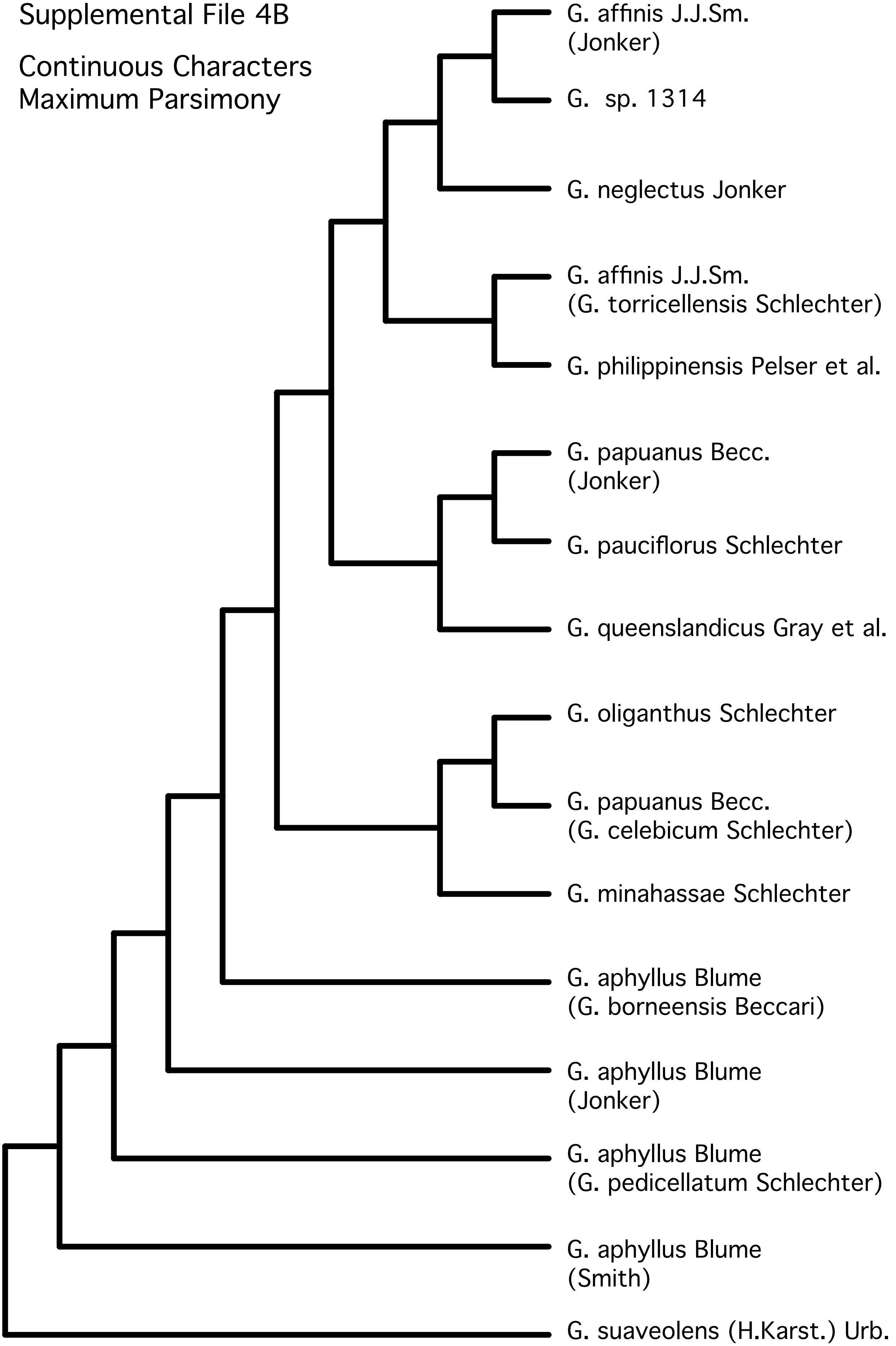

Supplemental File 4C

Categorical Characters

NJ tree

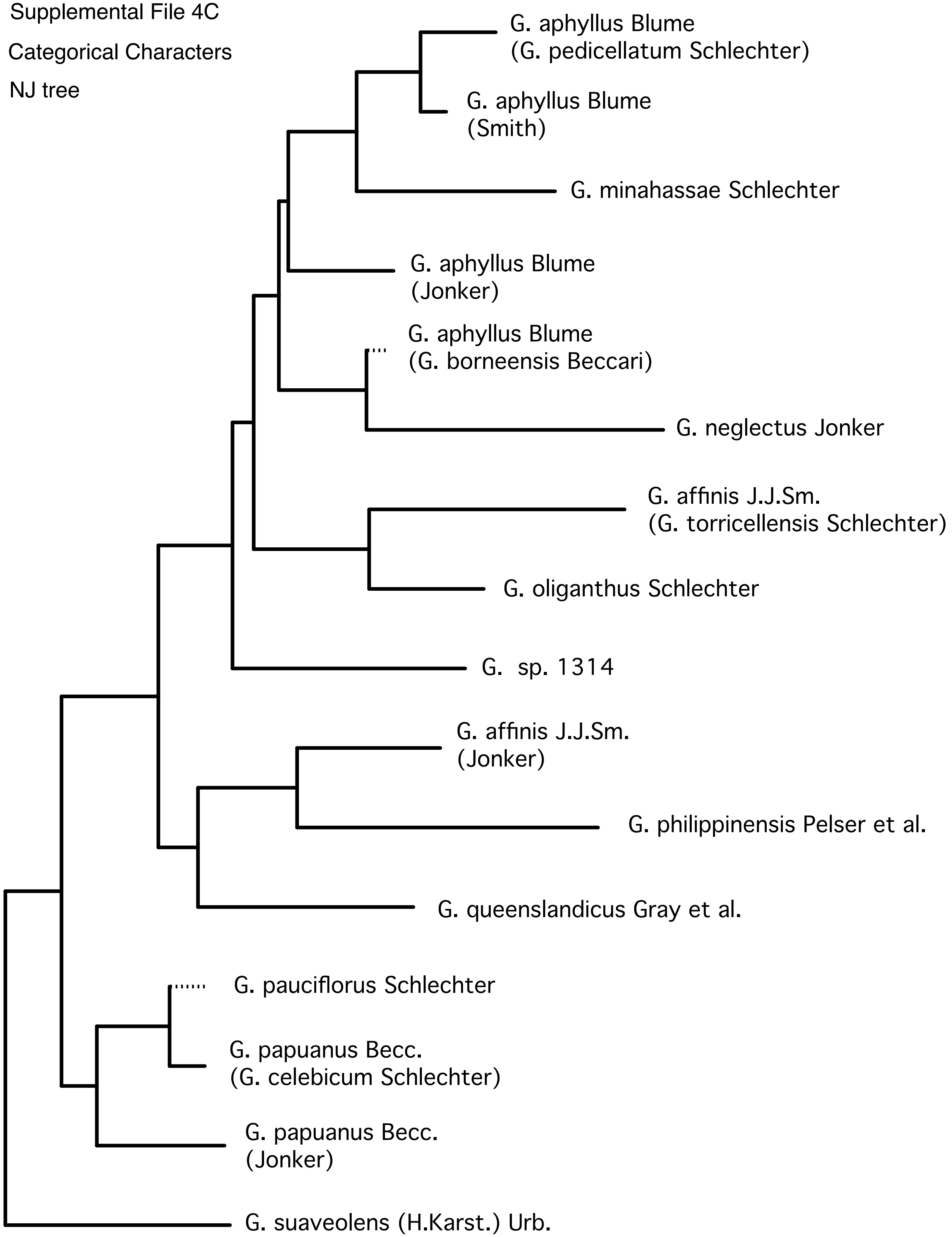

Supplemental File 4D

Categorical Characters
Maximum Parsimony

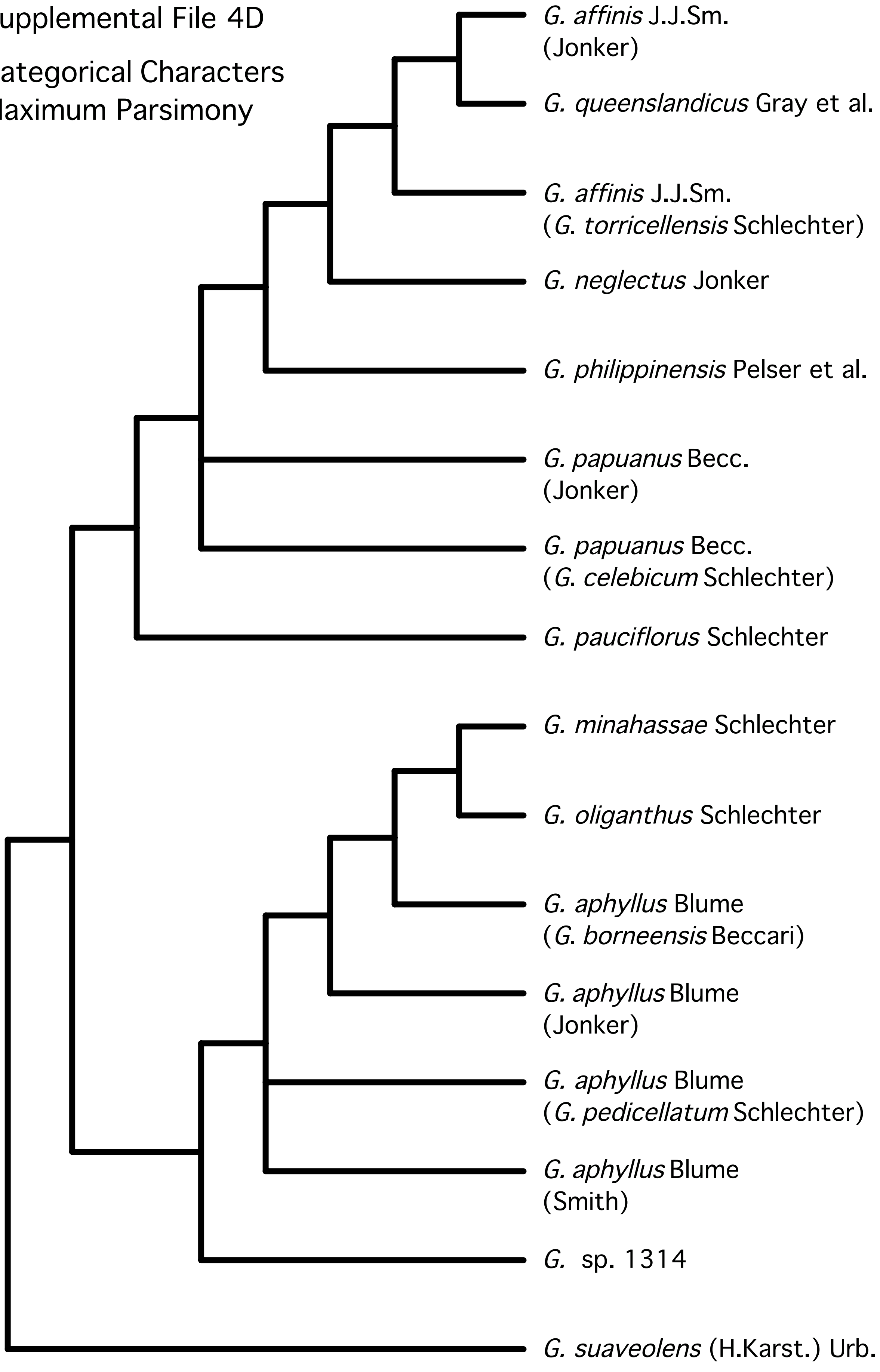

Supplement: Supplementary material 4 — Trees resulting from continuous and categorical characters analyzed separately using neighbor-joining (NJ) and maximum parsimony (MP) methods [file phytokeys-146-071-s004.pdf]
